# Fluctuation–response relations for integrate-and-fire models with an absolute refractory period

**DOI:** 10.1007/s00422-023-00982-9

**Published:** 2024-01-23

**Authors:** Friedrich Puttkammer, Benjamin Lindner

**Affiliations:** 1 Bernstein Center for Computational Neuroscience Berlin, Philippstr. 13, Haus 2, 10115 Berlin, Germany; 2 Physics Department of Humboldt University Berlin, Newtonstr. 15, 12489 Berlin, Germany

**Keywords:** Stochastic neuron models, Fluctuation–dissipation relations, Neural signal transmission, Spike-train analysis

## Abstract

We study the problem of relating the spontaneous fluctuations of a stochastic integrate-and-fire (IF) model to the response of the instantaneous firing rate to time-dependent stimulation if the IF model is endowed with a non-vanishing refractory period and a finite (stereotypical) spike shape. This seemingly harmless addition to the model is shown to complicate the analysis put forward by Lindner Phys. Rev. Lett. (2022), i.e., the incorporation of the reset into the model equation, the Rice-like averaging of the stochastic differential equation, and the application of the Furutsu–Novikov theorem. We derive a still exact (although more complicated) fluctuation–response relation (FRR) for an IF model with refractory state and a white Gaussian background noise. We also briefly discuss an approximation for the case of a colored Gaussian noise and conclude with a summary and outlook on open problems.

## Introduction

From a statistical physics point of view, neural systems are non-equilibrium systems that, if left alone, display pronounced fluctuations on all spatial scales and, if stimulated with signals from the environment, process these signals and respond to them. Two characteristic features of neural systems are thus the spontaneous activity under pure observation of the system and its response to time-dependent stimulation. Relations between the statistics of spontaneous fluctuations and the systematic response to external perturbations are known in statistical physics as fluctuation–dissipation theorems (Kubo 1966; Hänggi and Thomas 1982; Crisanti and Ritort 2003; Marconi et al. 2008) or, alternatively, as fluctuation–response relations (FRRs); their exploration for neural systems is so far limited to surprisingly few studies.

Starting at a strongly coarse-grained level, several groups have explored the relation and interactions between resting state activity and evoked brain activity in terms of electroencephalogram (EEG) and functional magnetic resonance imaging (fMRI) signals (He 2013; Huang et al. 2017) and found it to be highly non-trivial. Deco et al. (2023) fitted a stochastic model of coupled noisy Hopf-normal forms to neuroimaging data, for which fluctuation–dissipation relations are found to be obeyed if the model obeys detailed balance. (The model is considered only in its linearized form and the inherent nonlinearity of the noisy Andronov–Hopf-normal form (Lindner et al. 2009) does not play a role here.) Violations of the relation (deviations from the theorem averaged over all nodes of the network and perturbations) can be taken as a measure for the distance from equilibrium; remarkably, the authors find the strongest violation of the FDT if patients are engaged in the most demanding (the social) tasks. Sarracino et al. (2020) derived at a coarse-grained level a linearized stochastic version of the Wilson–Cowan model for neural populations and compare their results for the fluctuation and the response of this model to experimental magnetoencephalography recordings of resting activity and of activity evoked by visual stimuli; a recent extension of this approach (Nandi et al. 2023) is devoted to imbalanced networks of the Wilson–Cowan type. Cessac et al. (2021) have recently put forward a serious mathematical approach to the problem in terms of the probability densities of spike times and could derive approximate relations between spike response functions for a discrete-time leaky integrate-and-fire model neuron embedded in a network of similar units. Although this is certainly an important contribution, the suggested method seems hardly applicable to the standard multidimensional IF models in continuous time (which are known to yield the best performance in describing spiking of real cells in vivo, as demonstrated in a model competition, see Jolivet et al. (2008)). Finally, stochastic neural firing can be also often regarded as a stochastic oscillator, corresponding to a multidimensional nonlinear system of Langevin equations (e.g., for the Hodgkin–Huxley model with channel noise, see Fox (1997); Pu and Thomas (2020, 2021)), and in this interpretation it is interesting to note that a simple FRR can be obtained by a mapping those systems to a complex-valued variable related to eigenfunctions expansion for the associated probability density (Pérez-Cervera et al. 2023); with the new abstract variable, it is, however, not evident how to find direct connections to the response and fluctuation statistics of the spike train and the membrane voltage, i.e., to the observables which are directly accessible in experiment.

For an important class of stochastic neuron models, so-called integrate-and-fire (IF) models with Gaussian noise (see Burkitt (2006a, 2006b) for reviews), one of us derived relations between the susceptibility of the firing rate and the spontaneous spike statistics (Lindner 2022a, b) based on a novel combination of methods. For the standard leaky integrate-and-fire (LIF) model with white Gaussian noise, described by the stochastic differential equation

1 $$\begin{aligned} \frac{dv}{dt}=-v+\mu +s(t) +\sqrt{2D}\xi (t), \end{aligned}$$

complemented by the fire-and-reset rule that if $v(t)\ge v_T$ we register a spike at time $t=t_i$ and reset the voltage to $v(t)=v_R$, the susceptibility $\chi (\omega )$ of the spike train $x(t)=\sum \delta (t-t_i)$ with respect to the weak current signal *s*(*t*) in the above equation can be related the cross- and power spectra of the spike train and the subthreshold voltage *v*(*t*) as follows:

2 $$\begin{aligned} \chi _x(\omega )=\frac{(v_T-v_R)S_{xx}(\omega ) +(1+i\omega )S_{xv}(\omega )}{2D}. \end{aligned}$$

On the left-hand side, we have a statistics of the time-dependent mean value in response to a weak signal. On the right-hand side, we have statistics of the spontaneous fluctuations; hence, the relation constitutes a FRR. Lindner (2022a) confirmed the relation by numerical simulations; it was also exploited to derive an analytical expression for the cross-spectrum of the subthreshold membrane voltage and the spike train. Moreover, another exact FRR was derived for the more realistic and dynamically rich adaptive exponential integrate-and-fire model with colored Gaussian noise and confirmed by numerical simulation results.

The derivations by Lindner (2022a) became possible by the combination of two new ideas: (i) the reset of the voltage was formally incorporated into the voltage dynamics by adding a term involving the neuron’s spike train; (ii) an emerging noise-spike-train cross-correlation function was expressed by the susceptibility by virtue of the Furutsu–Novikov theorem (Furutsu 1963; Novikov 1965).

One weakness of the models studied by Lindner (2022a) is that they did not include an absolute refractory period. Integrate-and-fire models do not describe explicitly the action potential itself (Izhikevich 2007), so a minimal dead time for the generation of the next spike time should be the width of the spike itself (at least one millisecond and in many neurons substantially bigger than that). Although the addition of an absolute refractory period is often regarded as something trivial, it is not in the context of the FRRs (as will be revealed below). Related to this problem is that in experiments the subdivision of spike and subthreshold membrane voltage appears to be a rather artificial one—it would be much better to include a finite shape of the action potential into the voltage trace (even if this is only a stereotypical shape) when computing correlation functions of voltage and spike train.

In this paper we would like to generalize the FRRs derived by Lindner (2022a) to IF models with an absolute refractory period and a stereotypical spike shape. We achieve our goal for IF models driven by white Gaussian noise, for which an exact FRR can be derived. We also discuss the problems encountered when a colored noise is used and present an idea to come up with an approximate FRR for this case.

Our paper is organized as follows. In the next section we introduce the integrate-and-fire model that includes an absolute refractory period during which the voltage undergoes a prescribed stereotypical pulse shape; here we also derive some general relations for an IF model with an absolute refractory period and colored noise. In Sec. 3 we consider the simple example of an IF model driven by white noise for which we can derive an exact FRR. In Sec. 4 we turn to the case of a temporally correlated noise and derive an approximate FRR under the assumption that the noise correlation time is shorter than the mean interspike interval (ISI). We conclude in Sec. 5 with a short summary and a discussion of open problems.

## General model and preliminary calculations

We consider an IF model that is complemented by an absolute refractory period during which the voltage follows a stereotypical pulse shape $v_{\text {spike}}(t)$. Put differently, we let the subthreshold voltage evolve according to the usual integrator dynamics until it reaches the threshold $v_T$ at the firing time $t_i$. The set of these spike times *S* defines the main output of the neuron, the spike train $x(t)=\sum _{t_i\in S}\delta (t-t_i)$; differences between subsequent spike times $t_i-t_{i-1}$ are the interspike intervals (ISIs).

After each threshold crossing, we prescribe the spike shape during $[t_i,t_i+\tau _{\text {ref}}]$ by setting the time derivative as follows:

3 $$\begin{aligned} \dot{v}\!=\!{\left\{ \begin{array}{ll} \dot{v}_{\text {spike}}(t-t_i),\hspace{1.5em}\text {if }\exists \ t_i \in S \text { with }t_i\le t<t_i+\tau _{\text {ref}}\\ f(v)\!+\!\eta (t)\!+\!\varepsilon s(t),\hspace{1.5em}\text {otherwise.} \end{array}\right. } \end{aligned}$$

The voltage variable *v* represents the membrane voltage of the neuron, which includes now also (different to the usual modeling by integrate-and-fire neurons) the action potential. The process $\eta (t)$ is a Gaussian noise representing mainly the random synaptic input received by the neuron. The general function *f*(*v*) captures the deterministic aspects of the dynamics of *v*.

The function $\varepsilon s(t)$ is a weak time-dependent signal. For $\varepsilon = 0$ (no signal) we talk about the spontaneous activity of the neuron, which can be characterized by the steady-state firing rate $r_0=\left\langle x \right\rangle$ (the index 0 indicates vanishing signal strength), the power spectrum of the spike train *x*(*t*),

4 $$\begin{aligned} S_{xx}=\lim _{T\rightarrow \infty }\frac{\left\langle \tilde{x}\tilde{x}^* \right\rangle }{T},\;\; \tilde{x}=\int \limits _0^T dt e^{i\omega t} x(t), \end{aligned}$$

and other spectral measures such as the cross-spectrum between voltage and spike train

5 $$\begin{aligned} S_{vx}=\lim _{T\rightarrow \infty }\frac{\left\langle \tilde{v}\tilde{x}^* \right\rangle }{T},\;\; \tilde{v}=\int \limits _0^T dt e^{i\omega t} v(t). \end{aligned}$$

For a weak signal with $0<\varepsilon \ll 1$, we can consider how the instantaneous firing rate follows it in linear response

6 $$\begin{aligned} r(t)=\left\langle x(t) \right\rangle =r_0+\int \limits _{-\infty }^{t} dt' K_{xs}(t-t') s(t') \end{aligned}$$

or in Fourier domain

7 $$\begin{aligned} \tilde{r}=\chi _x(\omega ) \tilde{s}, \;\; \chi _x(\omega )=\int \limits _{0}^{\infty } dt\ K_{xs}(t) \end{aligned}$$

with the susceptibility $\chi _x(\omega )$ being the Fourier transform of the linear response function $K_{xs}(t)$. Our overall aim is to relate the statistics of spontaneous firing (no signal, $\varepsilon =0$) to the response properties, specifically the susceptibility $\chi (\omega )$, in a FRR.

**Fig. 1:**
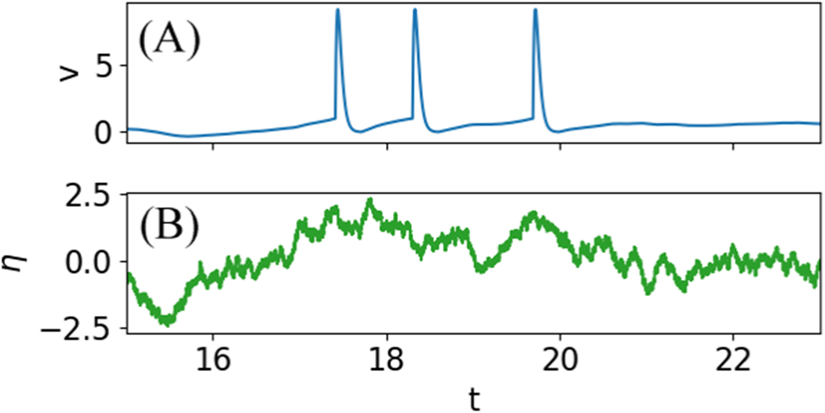
Time course of voltage (**A**) and Gaussian exponentially correlated noise (**B**) for a leaky IF model ($f(v)=\mu -v$) with an abstract spike shape $v_{\text {spike}}(t)=\kappa (t+t_0)e^{-\beta t}-\Delta v$ and no signal present ($\varepsilon =0$). Parameters: refractory period $\tau _{\text {ref}}=0.3$, noise variance $\sigma ^2=1$, correlation time $\tau _c=1$ (see Eq. (28)); spike parameters $\kappa =800$ and $\Delta v=0.01$ (dependent parameters $t_0\approx 12.6\times 10^{-4}$ and $\beta \approx 33.6$, see appendix Sec. 1)

We have to discuss the spike shape $v_{\text {spike}}(t)$. Generally, it has to obey the boundary conditions $v_{\text {spike}}(0)=v_T$ and $v_{\text {spike}}(\tau _{\text {ref}})=v_R$, thus resetting the voltage from the threshold value $v_T$ to the reset value $v_R$ within the duration of the refractory period $\tau _{\text {ref}}$. Apart from these constraints, our theory does not make any further assumptions on $v_{\text {spike}}(t)$. One option to choose the spike shape, if we are interested in modeling a specific cell for which voltage traces are available, would be to use an average over all action potentials in the voltage time series. A second option would be to use an analytically simple expression that still reflects the main features of the action potential, e.g., a simple alpha-type function

8 $$\begin{aligned} v_{\text {spike}}(t)=\kappa (t+t_0)e^{-\beta t}-\Delta v, \end{aligned}$$

see Fig. 1 for a stochastic simulation of the IF model with exponentially correlated Gaussian noise and this action potential shape. The parameters $\kappa , \beta , t_0,\Delta v$ can be related to the parameters $\tau _{\text {ref}},v_R$ and $v_T$ (see appendix Sec. 1) and also determine the height of the pulse. A third option is to ignore the specific shape of the action potential and to clamp the voltage to the reset potential for the duration of the refractory period, which can be written as follows

9 $$\begin{aligned} v_{\text {spike}}(t)=v_T-(v_T-v_R)\Theta (t),\;\;\; 0\le t\le \tau _\text {ref}. \end{aligned}$$

This formulation in terms of the Heaviside function might seem cumbersome. (After all, it simply means that $v_{\text {spike}}(t)\equiv v_R$ during the refractory state.) However, the incorporation of the Heaviside function leads to the reset of the voltage trajectory via the term $\dot{v}_{\text {spike}}(t)$, corresponding to the reset term $-(v_T-v_R)\sum _i \delta (t-t_i)$ that we would have in the previously studied case of an IF model without refractory period (Lindner 2022a). If we want to compare to the latter case, Eq. (9) is also more appropriate then having a non-vanishing pulse on top of the subthreshold voltage because the addition of a finite spike shape changes the cross-spectrum $S_{vx}(\omega )$ drastically. (It is no longer the cross-spectrum between the *sub*threshold voltage and the spike train but rather that of the full membrane voltage and the spike train.) We note that if we have experimental data in the form of a voltage time series *v*(*t*), it is always possible to create a purely subthreshold version of it, $v_\text {sub}(t)$ by setting the voltage to a fixed value during the refractory state. In this sense, the cross-spectrum $S_{vx}(\omega )$ calculated by Lindner (2022a) is a cross-spectrum between $v_\text {sub}(t)$ and the spike train. Whenever we contrast our new FRRs with the known one for $\tau _\text {ref}=0$, we will use a clamped voltage, Eq. (9), for better comparability.

In order to derive an FRR for the IF model with refractory period, we consider the dynamics in the absence of stimulation and, in analogy to the presentation in Lindner (2022a), we formally incorporate the reset of the trajectory into the voltage dynamics in a first step by means of the spike train *x*(*t*). Specifically, we introduce the refractory period indicator function

10 $$\begin{aligned} I_{\text {ref}}(t)&=\int \limits _{0}^{\tau _{\text {ref}}}d\tau 'x(t-\tau ')= \int \limits _{-\infty }^{\infty }d\tau 'B_{\tau _{ref}}(\tau ')x(t-\tau ')\nonumber \\&=B_{\tau _{ref}}*x(t)\nonumber \\&={\left\{ \begin{array}{ll} 1&{}\text {if }\exists \ t_i\in S\text { with }t_i\le t<t_i+\tau _{\text {ref}}\\ 0&{}\text {otherwise} \end{array}\right. } \end{aligned}$$

Here the asterisk denotes a convolution, and we have introduced the box-car function $B_{\tau _{\text {ref}}}$ given by Heaviside functions as $B_{\tau _{\text {ref}}}(t)=\Theta (t)\Theta (\tau _{\text {ref}}-t)$. This function is used to subtract the regular dynamics $f(v)+\eta (t)$ during the refractory period. Furthermore, we add the prescribed dynamics during the refractory period, using

11 $$\begin{aligned}&\int \limits _{0}^{\tau _{\text {ref}}}d\tau 'x(t-\tau ')\dot{v}_{\text {spike}}(\tau ')\nonumber \\&={\left\{ \begin{array}{ll} \dot{v}_{\text {spike}}(t-t_i)&{}\text {if }\exists \ t_i\in S\text { with }t_i\le t<t_i+\tau _{\text {ref}}\\ 0&{}\text {otherwise} \end{array}\right. } \end{aligned}$$

Finally, we use that during the refractory period after a spike at $t_i$ we have $f(v(t))=f(v_{\text {spike}}(t-t_i))$, because of the prescribed dynamics. Hence,

12 $$\begin{aligned}&f(v(t))I_{\text {ref}}(t)\nonumber \\&={\left\{ \begin{array}{ll} f(v_{\text {spike}}(t-t_i))&{}\text {if }\exists \ t_i\in S\text { with }t_i\le t<t_i+\tau _{\text {ref}}\\ 0&{}\text {otherwise} \end{array}\right. } \nonumber \\&=\int \limits _{0}^{\tau _{\text {ref}}}d\tau 'x(t-\tau ')f(v_{\text {spike}}(\tau ')) \end{aligned}$$

in analogy to Eq. (11), leading to the following formal expression for the voltage dynamics

13 $$\begin{aligned} \dot{v}=&\ f(v)+\eta (t)-\eta (t)I_{\text {ref}}(t)\nonumber \\&\ \hspace{-1em}+\int \limits _{0}^{\tau _{\text {ref}}}d\tau ' x(t-\tau ')[\dot{v}_{\text {spike}}(\tau ')-f(v_{\text {spike}}(\tau '))]. \end{aligned}$$

**Fig. 2:**
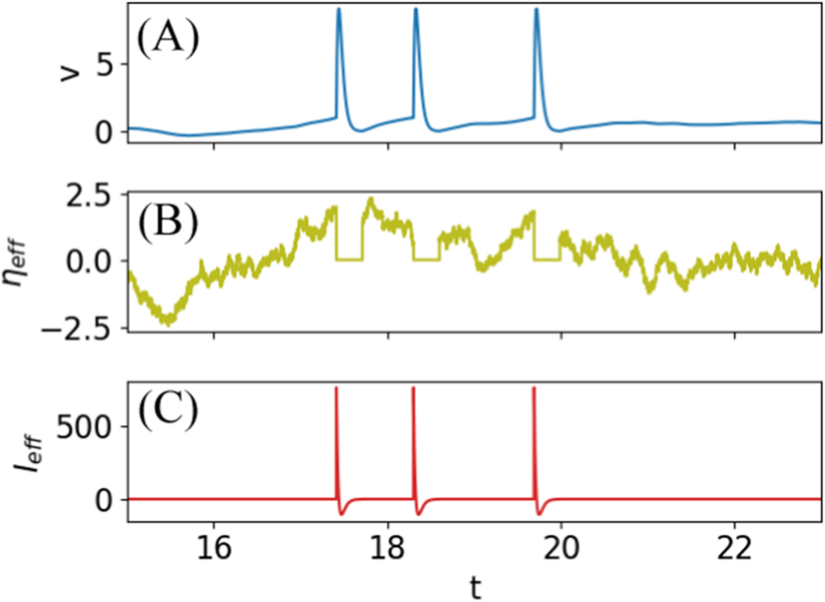
Illustration of auxiliary functions $\eta _{\text {eff}}(t)$ and $I_{\text {eff}}(t)$. Voltage time course (**A**) as in Fig. 1(A); effective noise seen outside the refractory periods (**B**) and the term enforcing the stereotypical spike shape (**C**). Leaky IF model with the abstract spike shape $v_{\text {spike}}(t)=\kappa (t+t_0)e^{-\beta t}-\Delta v$ and exponentially correlated Gaussian noise (see Eq. (28)). The parameters are the same as in Fig. 1

This can be interpreted as a voltage dynamics resulting from an effective noise $\eta _{\text {eff}}$, which is turned off during the refractory period

14 $$\begin{aligned} \eta _{\text {eff}}(t)=\eta (t)[1-I_{\text {ref}}(t)], \end{aligned}$$

and an effective input current $I_{\text {eff}}$ (second line of Eq. (13)) that implements the stereotypical spike shape $v_{\text {spike}}(t)$ and subtracts the deterministic part of the dynamics. Figure 2 illustrates these auxiliary functions of the system with a refractory state and a stereotypical spike shape.

In analogy to the procedure by Lindner (2022a) (see in particular the preprint version by Lindner (2022b)), we use a time-domain version of the Rice method (see Rice (1944) and (Risken 1984), Sec. 3.2.3 for a simple exposition) to establish a relation among certain correlation functions. Specifically, we take Eq. (13) at a lagged time argument $t+\tau$, multiply with the spike train *x*(*t*) and average, yielding

15 $$\begin{aligned} \frac{d C_{xv}}{d\tau }=&\ C_{xf(v)}(\tau )- I_{\text {spike}}*C_{xx}(\tau )\nonumber \\&+C_{x\eta }(\tau )-\int \limits _{0}^{\tau _{\text {ref}}}d\tau '\langle x(0) x(\tau -\tau ')\eta (\tau )\rangle +C_1. \end{aligned}$$

where we used that

16 $$\begin{aligned}&\int \limits _{0}^{\tau _{\text {ref}}}d\tau 'x(t-\tau ')[\dot{v}_{\text {spike}} (\tau ')-f(v_{\text {spike}}(\tau '))]\nonumber \\&=I_{\text {spike}}*x(t) \end{aligned}$$

with the effective input current associated to an individual spike $I_{\text {spike}}(t)=B_{\tau _{\text {ref}}}(t)[\dot{v}_{\text {spike}}(t)-f(v_{\text {spike}}(t))]$. The constant $C_1$ is given by

17 $$\begin{aligned} C_1=r_0\left[ \langle f(v)\rangle -r_0\int \limits _0^{\tau _{\text {ref}}}d\tau '\dot{v}_{\text {spike}}(\tau ')-f(v_{\text {spike}}(\tau '))\right] .\nonumber \\ \end{aligned}$$

In order to arrive at the desired relation between spontaneous statistics and response statistics (similar to Eq. (2)), we next Fourier transform Eq. (15), crossing over from (auto- and cross-)correlation functions to (power and cross-)spectra. We then use for the cross-spectrum between the Gaussian input noise and the output spike train (which is a functional of the former) the Furutsu–Novikov theorem (Furutsu 1963; Novikov 1965)

18 $$\begin{aligned} S_{\eta x}(\omega )=\chi _x(\omega )S_{\eta \eta }(\omega ), \end{aligned}$$

according to the argument presented by Lindner (2022a). This brings the susceptibility into the relation. However, there is now a novel problem, in the form of the triple correlation in $\langle x(0)x(\tau -\tau ')\eta (\tau )\rangle$, which cannot be easily simplified. The relation that we obtain so far by the steps outlined above reads for $\omega >0$

19 $$\begin{aligned} \chi _x= & {} \frac{i\omega S_{xv}-S_{xf(v)}+\mathcal {F} (I_{\text {spike}}))S_{xx}}{S_{\eta \eta }}\nonumber \\{} & {} \hspace{-0.5em}+\frac{1}{S_{\eta \eta }} \int \limits _{-\infty }^\infty d\tau e^{i\omega \tau } \int \limits _{0}^{\tau _{\text {ref}}}d \tau '\langle x(0)x(\tau -\tau ')\eta (\tau )\rangle \end{aligned}$$

Unfortunately, we have not been able to derive an explicit expression for the last term in this preliminary relation in the general case. However, before proceeding to special cases, we can recast the triple correlation using the identity

20 $$\begin{aligned} \langle x(0)x(\tau -\tau ')\eta (\tau )\rangle =\left[ C_{xx}(\tau -\tau ')+r_0^2\right] \langle \eta (\tau )\rangle _{0,\tau -\tau '}\nonumber \\ \end{aligned}$$

which is derived in the appendix, Sec. 3. Here $\langle \cdot \rangle _{t_1,t_2}$ denotes the average conditioned on the certain occurrence of spike times at $t_1$ and $t_2$.

We next consider in Sec. 3 the case of uncorrelated (white) noise, for which the term in question can be shown to vanish identically, leading to an exact FRR. In Sec. 4 we then develop an approximation for the case of a temporally correlated (colored) noise.

## IF model with white Gaussian noise

We consider the special case of a white Gaussian noise $\eta (t)=\sqrt{2D}\xi (t)$ with $C_{\xi \xi }(t)=\delta (t)$ (and thus $S_{\eta \eta }=2D$). In this scenario, the triple correlation $\langle x(0)x(\tau -\tau ')\eta (\tau )\rangle$ vanishes because in the equivalent expression Eq. (20)

21 $$\begin{aligned} \langle \xi (\tau )\rangle _{0,\tau -\tau '}=\langle \xi \rangle =0,\quad 0<\tau '<\tau _{\text {ref}} \end{aligned}$$

This, fortunately, implies that the problematic contribution (the second line in Eq. (19)) vanishes. To see why Eq. (21) holds true, note that this equation contains the conditional average of the white Gaussian noise $\langle \xi (\tau )\rangle _{0,\tau -\tau '}$ given spiking at $t=0$ and $t=\tau -\tau '$. In the context of the IF model, specific spike times are caused by the specific noise realization, and we can use Bayes’ theorem to evaluate the conditional mean value by means of the conditional probability density of the spike times given the noise realization:

22 $$\begin{aligned} \langle \xi (\tau )\rangle _{0,\tau -\tau '}= & {} \int d\hat{\xi }\ \hat{\xi }\ P(\hat{\xi }, \tau |t_i=0,t_j=\tau -\tau ')\nonumber \\= & {} \int d\hat{\xi }\ \hat{\xi }\ \frac{P(t_i=0,t_j=\tau -\tau '|\hat{\xi },\tau ) P(\hat{\xi }, \tau )}{P(t_i=0,t_j=\tau -\tau ')}\nonumber \\= & {} \int d\hat{\xi }\ \hat{\xi }\ \frac{P(t_i=0,t_j=\tau -\tau ') P(\hat{\xi }, \tau )}{P(t_i=0,t_j=\tau -\tau ')}\nonumber \\= & {} \int d\hat{\xi }\ \hat{\xi }\ P(\hat{\xi }, \tau )=\langle \xi \rangle =0 \end{aligned}$$

In the third line we used that, for our specific construction, the probability for a spike at $t_i=0$ and another one at $t_j=\tau -\tau '$ is independent of the noise value $\hat{\xi }$ at $\tau$ because (i) the time argument $\tau$ falls into a refractory period and thus $\hat{\xi }$ does not affect the voltage dynamics and (ii) it is also uncorrelated to noise values outside the refractory state that *do* affect the voltage dynamics and the spike timing. Hence, the conditional mean value is equal to the unconditional mean value over the white noise, which vanishes. In consequence, Eq. (19) becomes an exact FRR

23 $$\begin{aligned} \chi _x(\omega )=&\ \frac{1}{2D}\big [i\omega S_{xv}(\omega )-S_{xf(v)}(\omega )+\mathcal {F} \left( I_{\text {spike}}\right) S_{xx}(\omega )\big ] \end{aligned}$$

Specifically for a white noise-driven leaky IF (LIF) model with $f(v)=\mu -v$, (for which then $S_{xf(v)}(\omega )=-S_{xv}(\omega )$), the FRR reads

24 $$\begin{aligned} \chi _x(\omega )=\ \frac{[1+i\omega ] S_{xv}+\mathcal {F} \left( I_{\text {spike}}^{\text {LIFM}}\right) S_{xx}}{2D} \end{aligned}$$

where $I_{\text {spike}}^{LIFM}=B_{\tau _{\text {ref}}} [\mu -v_\text {spike}-\dot{v}_{\text {spike}}]$. We note that for the abstract spike shape, Eq. (8), illustrated in Fig. 1, the Fourier transform can be analytically calculated and is given in the appendix [see Sec. 2, Eqs. (42) and (44)] together with relations between the parameters $\kappa , \beta , t_0$ and $\Delta v$ to ensure that $v_{\text {spike}}(0)=v_T$ and $v_{\text {spike}}(\tau _\text {ref})=v_R$. Alternatively, as discussed in Sec. 2 we may ignore the effect of the stereotypical spike shape by clamping the voltage to the reset value according to Eq. (9). In this case, the Fourier transform of the refractory term reads

25 $$\begin{aligned} \mathcal {F}\left( I_{\text {spike}}^{\text {LIFM}}\right) (\omega ) =\frac{\mu -v_R}{i\omega }\left[ e^{i\omega \tau _{\text {ref}}}-1\right] +v_R-v_T \end{aligned}$$

**Fig. 3:**
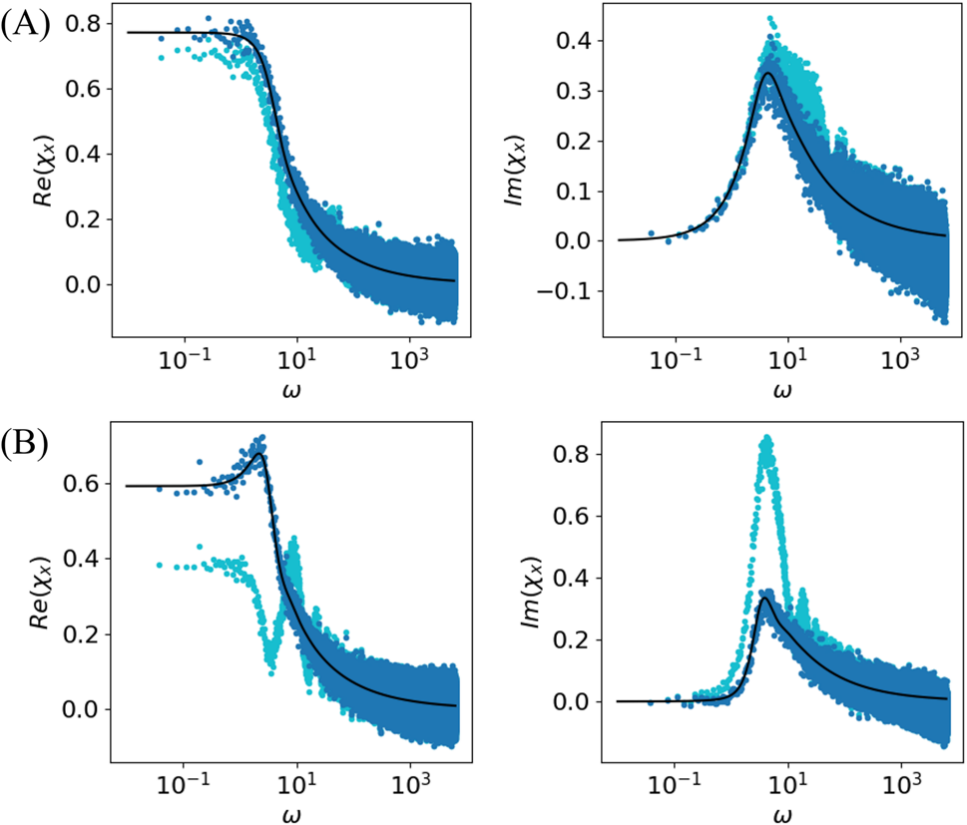
Susceptibility $\chi _x$ can be computed from the spontaneous statistics via the new FRR but not when the refractory period is neglected. Analytical expression for the susceptibility $\chi _x$ (Lindner and Schimansky-Geier 2001) (solid black lines) compared to the prediction of the new FRR Eq. (24) (dark blue) and the previously known FRR for the non-refractory case (Lindner 2022a) (light blue) based on numerical results from a simulation of $N=10^3$ realizations of the LIF model with time step $\Delta t=10^{-5}$ and time window $T={2}^{24}\Delta t\approx 167$. Parameters: $\mu =0.8$, $\tau _{\text {ref}}=0.1\ll 1/r_0\approx 2.8$ (**A**) and $\tau _{\text {ref}}=0.5<1/r_0\approx 3.2$ (**B**)

For the LIF model with an absolute refractory period, analytical expressions for the susceptibility $\chi _x$ and the spike train power spectrum $S_{xx}$ have been derived (Lindner and Schimansky-Geier 2001; Lindner et al. 2002). We can now test Eq. (24) in two ways. First of all, we can measure the spontaneous statistics for an LIF model with clamped potential during the refractory state, i.e., we apply Eq. (9) and just take into account the subthreshold voltage. From the spontaneous spectra $S_{xx}(\omega )$ and $S_{xv}(\omega )$, we may then predict the susceptibility via Eq. (24) and compare it to the analytically known expression. This is illustrated in Fig. 3 and confirms the relation for two different values of $\tau _{\text {ref}}$. We note that increasing the refractory period is generally detrimental to the signal transmission: The overall magnitude of the susceptibility goes down by increasing the dead time after each spike during which the neuron ’does not see’ the stimulus. Because a large value of the refractory period makes the spiking more regular, the reduced susceptibility may develop a peak around the firing rate (here around $\omega \approx 2\pi r_0$), which becomes apparent in Fig. 3B where $\tau _\text {ref}=0.5$.

However, what is really the role of the finite refractory period in the new FRR Eq. (23). In order to access the effect of the refractory period, we use the relation for a neuron with vanishing $\tau _\text {ref}$ derived by Lindner (2022a) [or, equivalently, Eq. (24) for $\tau _\text {ref}=0$] to extract the susceptibility from the spontaneous activity in the presence of a refractory period (cyan lines in Fig. 3); this procedure leads to a small but significant error when the refractory period is small ($\tau _\text {ref}=0.1$ accounting for 3.5% of the mean ISI; cyan lines in Fig. 3(A)), but it results in a strongly erroneous estimation of the susceptibility when the refractory period has an intermediate value ($\tau _\text {ref}=0.5$ accounting for 16% of the mean ISI; cyan lines in Fig. 3(B)).

**Fig. 4:**
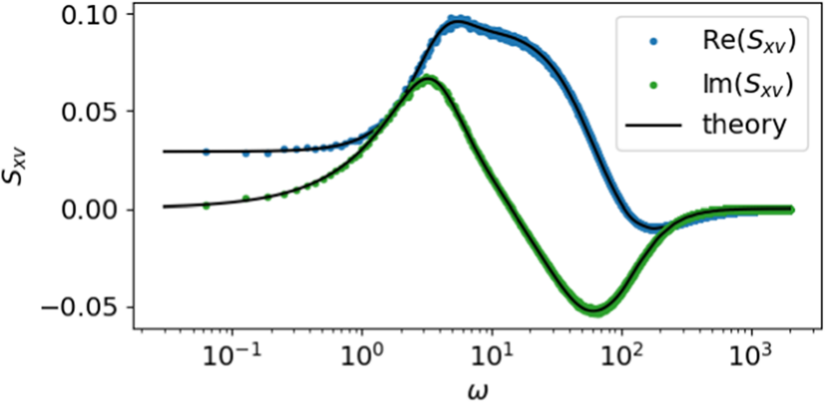
The new FRR makes an accurate prediction of the cross-spectrum $S_{xv}$. Comparison of the prediction of the cross-spectrum $S_{xv}$ of the new FRR (solid black line) with numerical results (blue and green dots) for the LIF model with Gaussian uncorrelated noise and an abstract spike shape $v_{\text {spike}}(t)=\kappa (t+t_0)e^{-\beta t}-\Delta v$. Simulation parameters: time step $\Delta t=10^{-4}$, time window $T=100$; model parameters: $\mu =0.8$, $\tau _{\text {ref}}=0.1\ll 1/r_0\approx 2.8$; spike parameters: $\kappa =2500$ and $\Delta v=0.01$ (dependent parameters $t_0\approx 4\times 10^{-4}$ and $\beta \approx 101.3$, see appendix Sec. 1)

We can validate Eq. (24) in a second way. All spectral statistics in the equation are analytically known except for the cross-spectrum of spike train and voltage $S_{xv}$. We can easily solve for this function and obtain

26 $$\begin{aligned} S_{xv}(\omega )=\frac{2D\chi _x(\omega )-\mathcal {F} \left( I_{\text {spike}}^{\text {LIFM}}\right) S_{xx} (\omega )}{1+i\omega } \end{aligned}$$

We measure the cross-spectrum in the stochastic simulations in the version with a finite spike shape, Eq. (8), and compare its real (blue) and imaginary (green) parts in Fig. 4 to the analytical expression in Eq. (26) (solid black lines). The agreement is excellent and can be regarded as another confirmation of the FRR, especially in the version with a finite stereotypical pulse shape.

In the form of Eq. (26) the FRR can be intuitively interpreted. Its r.h.s. reflects that there are two sources of correlation between voltage *v*(*t*) and spike train *x*(*t*). For once, both voltage and spike train respond to the noise $\sqrt{2D}\xi (t)$ and are correlated because of this common drive, which leads to the first contribution, $2D\chi _x(\omega )$, in the numerator of the fraction. Secondly, the voltage is subject to a stereotypical input $I_{\text {spike}}$ after each threshold crossing recorded in the spike train. Hence, the voltage is in part correlated to the spike train *x*(*t*) as *x*(*t*) is correlated to itself, which is reflected in the second contribution in the numerator, $\mathcal {F}\left( I_{\text {spike}}^{\text {LIFM}}\right) S_{xx}(\omega )$.

## IF model with colored Gaussian noise

We can generate a low-pass-filtered (colored) noise by simulating an additional stochastic differential equation, the Ornstein–Uhlenbeck process

27 $$\begin{aligned} \tau _c \dot{\eta }=-\eta +\sqrt{2\sigma ^2 \tau _c} \xi (t), \end{aligned}$$

where $\tau _c$ and $\sigma ^2$ are the correlation time and variance of the noise, respectively. The stationary process has a Lorentzian power spectrum and an exponential correlation function:

28 $$\begin{aligned} S_{\eta \eta }(\omega )=\frac{2\sigma ^2\tau _c}{1 + (\tau _c\omega )^2}, \;\; C_{\eta \eta }(t)=\sigma ^2e^{-|t|/\tau _c}. \end{aligned}$$

The incorporation of a colored noise by an additional stochastic differential equation is called Markovian embedding (Hänggi and Jung 1995) [other, more general examples of that are discussed, for instance by Vellmer and Lindner (2019)]. We emphasize that the following derivation does not rely on the specific form of the colored noise given by the Ornstein–Uhlenbeck process Eq. (27) but only on its Gaussianity and conditions for the correlation time (see below).

The temporal correlations of the noise complicate the calculation of the triple correlation, i.e., of Eq. (20) that we quote here again

29 $$\begin{aligned} \langle x(0)x(\tau -\tau ')\eta (\tau )\rangle = \left[ C_{xx}(\tau -\tau ')+r_0^2\right] \langle \eta (\tau )\rangle _{0,\tau -\tau '}\nonumber \\ \end{aligned}$$

to discuss an approximation for the r.h.s. For large $\tau$ and short refractory period durations $\tau _{\text {ref}}$

30 $$\begin{aligned} \langle \eta (\tau )\rangle _{0,\tau -\tau '}\approx \langle \eta (\tau )\rangle _{\tau -\tau '},\quad 0<\tau '<\tau _{\text {ref}}. \end{aligned}$$

Plausibly, $\tau$ has to be large compared to the characteristic correlation time $\tau _c$ of the noise process for this approximation to hold. To justify the use of Eq. (30) in Eq. (29), we note that the other factor in the product, $C_{xx}(\tau -\tau ')+r_0^2$ is close to zero if $|\tau -\tau '|\ll 1/r_0$ – the probability of two spikes in short succession vanishes or is rather low because of absolute and relative refractoriness. We insert Eq. (30) into Eq. (29), yielding

31 $$\begin{aligned}&\langle x(0)x(\tau -\tau ')\eta (\tau )\rangle&\approx \left[ C_{xx}(\tau -\tau ')+ r_0^2\right] \langle \eta (\tau )\rangle _{\tau -\tau '},\nonumber \\{} & {} \hspace{6em} 0<\tau '<\tau _{\text {ref}} \end{aligned}$$

As shown in the Appendix, Sec. 4, the last factor can be further approximated by

32 $$\begin{aligned} \langle \eta (\tau )\rangle _{\tau -\tau '}\!=\!\langle \eta (0)\rangle _{-\tau '}\approx & {} \frac{1}{\tau _{\text {ref}}} \int \limits _0^{\tau _{\text {ref}}}d\tau ''\langle \eta (0)\rangle _{-\tau ''}\!=\!\langle \eta \rangle _{\text {ref}}, \nonumber \\{} & {} 0<\tau '<\tau _{\text {ref}} \end{aligned}$$

for short refractory periods. In the last step we defined the average value of the noise during the refractory period, $\langle \eta \rangle _{\text {ref}}$.

By a stationary average of the IF dynamics Eq. (13) with $d\langle v\rangle /dt=0$ and use of $\langle x(-\tau ')\eta (0)\rangle =r_0\langle \eta (0)\rangle _{-\tau '}$ (see Appendix Sec. 3, Eq. (49)), we find

33 $$\begin{aligned} \langle \eta \rangle _{\text {ref}}=\frac{1}{\tau _{\text {ref}}}\left[ \frac{\langle f(v)\rangle }{r_0} \!-\!\!\int \limits _0^{\tau _{\text {ref}}}d\tau 'f(v_{\text {spike}}(\tau '))-\dot{v}_{\text {spike}}(\tau ')\right] . \end{aligned}$$

Using this expression in Eq. (32) and Eq. (31) culminates in the following approximate expression for the triple correlation

34 $$\begin{aligned}&\langle x(0)x(\tau -\tau ')\eta (\tau )\rangle \approx \ \left[ C_{xx}(\tau -\tau ')+r_0^2\right] \times \nonumber \\&\frac{1}{\tau _{\text {ref}}}\left[ \frac{\langle f(v)\rangle }{r_0}- \int \limits _0^{\tau _{\text {ref}}}d\tau 'f(v_{\text {spike}}(\tau '))-\dot{v}_{\text {spike}}(\tau ')\right] . \end{aligned}$$

If we insert this expression into Eq. (19), we obtain the approximate FRR

35 $$\begin{aligned}&\chi _x(\omega )\approx \ \frac{1}{S_{\eta \eta }(\omega )} \Bigg [i\omega S_{xv}(\omega )-S_{xf(v)}(\omega )\nonumber \\&+\mathcal {F}(I_{\text {spike}})(\omega )S_{xx}(\omega )+ \frac{1}{\tau _{\text {ref}}}\left\{ \frac{\langle f(v)\rangle }{r_0}\right. \nonumber \\&\left. -\int \limits _0^{\tau _{\text {ref}}}d\tau 'f (v_{\text {spike}}(\tau '))-\dot{v}_{\text {spike}} (\tau ')\right\} \tilde{B}_{\tau _{\text {ref}}}(\omega )S_{xx}(\omega )\Bigg ], \end{aligned}$$

where $\tilde{B}_{\tau _{\text {ref}}}(\omega )=\mathcal {F}(B_{\tau _{\text {ref}}}) (\omega )=\frac{1}{i\omega }\left[ e^{i\omega \tau _{\text{ ref }}}-1\right]$. Again, this relation connects the response statistics in the case of stimulation to the statistics of spontaneous firing and the noise spectrum $S_{\eta \eta }$. Note that the dependence of the susceptibility on the refractory period is more complicated than may appear at the first glance: For instance, all fluctuation spectra, $S_{xx}, S_{xv}$ and $S_{xf(v)}$, depend on $\tau _\text {ref}$. As in the case of white noise (see the discussion of Fig. 3), we expect that the overall susceptibility is reduced by increasing $\tau _\text {ref}$, simply because we increase the period of time in which the stimulus cannot affect the neural dynamics. (The detailed effects of the refractory period on the spectral structure of the susceptibility for a colored noise-driven IF neuron are certainly worth an additional study.)

One way to test this FRR is to measure both the response and the spontaneous power and cross-spectra and to solve the above relation for the noise spectrum. In the special case of an LIF neuron, the resulting estimate of the intrinsic noise spectrum reads

36 $$\begin{aligned}&S_{\eta \eta }(\omega )\approx \ \frac{1}{\chi _x(\omega )}\Bigg [(1+i\omega ) S_{xv}(\omega )+\nonumber \\&\mathcal {F}\left( I_{\text {spike}}^{\text {LIFM}}\right) (\omega )S_{xx}(\omega )+ \frac{1}{\tau _{\text {ref}}}\left\{ \frac{\mu -\langle v\rangle }{r_0}\right. \nonumber \\&\left. -\int \limits _0^{\tau _{\text {ref}}}d\tau '[\mu -v_{\text {spike}}(\tau ')- \dot{v}_{\text {spike}}(\tau ')]\right\} \tilde{B}_{\tau _{\text {ref}}}(\omega )S_{xx}(\omega )\Bigg ]. \end{aligned}$$

**Fig. 5:**
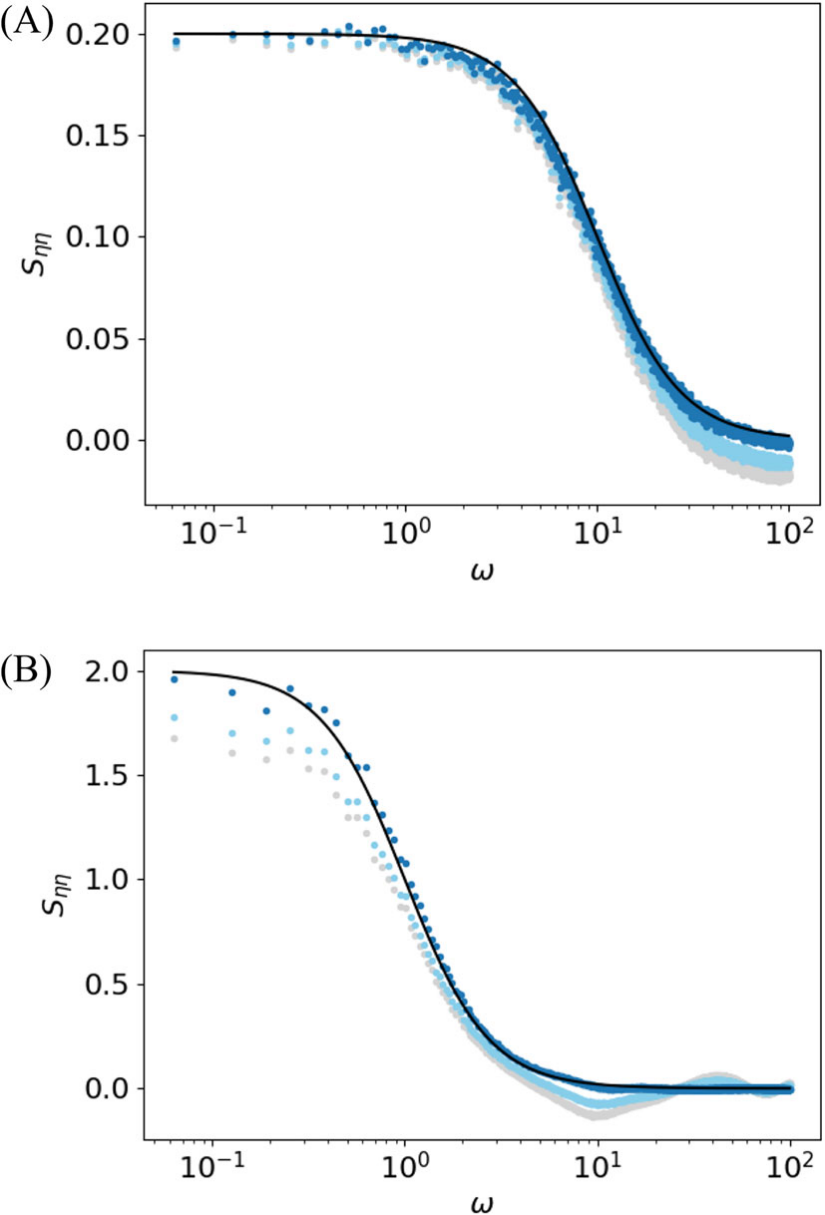
Approximate FRR for LIF model with colored noise predicts the intrinsic noise spectrum correctly. We compare the prediction of the new approximate FRR Eq. (36) (dark blue dots) to the exact theory Eq. (28). LIF neuron with $\mu =0.8$ driven by an Ornstein–Uhlenbeck noise with variance $\sigma ^2=1$ and a correlation time of $\tau _c=0.1$ (short compared to the mean ISI $\tau _c\approx 1/(40 r_0)$) in (**A**) and $\tau _c=1$ (a third of the mean ISI) in (**B**). In both cases we adapt the refractory period to be much smaller than the correlation time by setting $\tau _\text {ref}=\tau _c/10$

We check this for an LIF model with a clamped voltage during the refractory period (according to Eq. (9)) subject to an Ornstein–Uhlenbeck noise with the prescribed power spectrum Eq. (28); indeed, Eq. (36) (dark blue dots in Fig. 5) is for all frequencies on top of the exact result (solid black line) both for short (A) and intermediate (B) correlation time $\tau _c$. More examples are discussed by Puttkammer (2023).

We can further access the validity of our approximation by comparing to two more naive approximations. First of all, we can compare to the prediction from the exact FRR for the case of a colored noise-driven LIF model with vanishing refractory period [this corresponds to the second model considered by Lindner (2022a) with the nonlinear function of an LIF neuron and vanishing adaptation, i.e., $f(v)=\mu -v$ and $\Delta _a=0$ in Eq. (10) by Lindner (2022a)]:

37 $$\begin{aligned} S_{\eta \eta }(\omega )\approx \frac{[1+i\omega ]S_{xv}(\omega )+[v_T-v_R]S_{xx}(\omega )}{\chi _x(\omega )}. \end{aligned}$$

This approximation is shown by gray dots in the two panels of Fig. 5 and shows systematic deviations from the true spectrum: for short correlation times, deviations are limited to high frequencies; for a ’more colored’ noise, we observe stronger deviations also at low frequencies and also oscillations and unphysical negative values at higher frequencies.

Alternatively, we can take the formula from the previous section for a white noise-driven LIF neuron, Eq. (24), replace ad hoc the white noise spectrum 2*D* by the colored-noise spectrum $S_{\eta \eta }(\omega )$, and solve the resulting equation for the latter function, yielding

38 $$\begin{aligned} S_{\eta \eta }(\omega )\approx \frac{(1+i\omega ) S_{xv}\!+\!\mathcal {F}(I_\text {spike}^{LIF})S_{xx}}{\chi _x}. \end{aligned}$$

This approximation is closer to the true spectrum but also shows systematic deviations (see light blue dots in Fig. 5). Hence, our approximate FRR Eq. (35), specifically as recast in Eq. (36), is more appropriate for estimating the intrinsic noise spectrum than the two naive approximations that either neglect the effects of the refractory period (i.e., Eq. (37)) or the non-vanishing correlation time of the noise (i.e., Eq. (38)).

To interpret Eq. (36), we solve for the cross-spectrum $S_{xv}$ and reintroduce the average value of the noise during the refractory period $\langle \eta \rangle _{\text {ref}}$ according to Eq. (33)

39 $$\begin{aligned} S_{xv}(\omega )=&\ \frac{1}{1+i\omega }\Big [\chi _x(\omega )S_{\eta \eta }(\omega )- \mathcal {F}\left( I_{\text {spike}}^{\text {LIFM}}\right) S_{xx}(\omega )\nonumber \\&-\langle \eta \rangle _{\text {ref}} \tilde{B}_{\tau _{\text {ref}}}(\omega )S_{xx}(\omega )\Big ] \end{aligned}$$

The r.h.s. captures once again the different sources of correlation between voltage *v*(*t*) and spike train *x*(*t*). Comparing Eq. (39) with the white noise equivalent Eq. (26), we can tell that the two sources of correlation discussed at the end of Sec. 3 are still present. However, Eq. (39) includes a third contribution (second line), that is due to the non-vanishing mean value $\langle \eta \rangle _{\text {ref}}$ of the noise during the refractory period when the noise is temporally correlated.

## Summary and open problems

We have extended the FRRs developed by Lindner (2022a) for integrate-and-fire models with a non-vanishing refractory period and a finite pulse shape. For many standard statistics of interest, taking into account an absolute refractory period is straightforward. For instance, if we are interested in the mean ISI, we would have to add $\tau _\text {ref}$ to the mean ISI in the absence of a refractory period to obtain its correct value—a quite trivial operation! Also for more complicated statistics, as for instance the spike train power spectrum and the susceptibility of the rate modulation, the incorporation of the refractory period is not difficult [see, e.g., Lindner (2002) and, for colored noise, Vellmer and Lindner (2019)]. However, if it comes to deriving an FRR for an IF model, significant efforts are needed to incorporate the absolute refractory state and a finite pulse shape in the IF model’s equation. Here we achieved this incorporation and computed new terms arising from the refractory state and the pulse shape (i) exactly in the case of a white Gaussian intrinsic noise and (ii) approximately in the case of a shortly correlated (colored) Gaussian noise.

Our results show that the incorporation of a non-vanishing value of $\tau _\text {ref}$ makes a big difference if we want to apply the FRR. In case of a white-noise-driven leaky IF model, for instance, we saw that the susceptibility of the neuron (its response to an external stimulus) can be very accurately predicted from the spontaneous activity and its spectral statistics alone. In contrast, using the FRR for $\tau _\text {ref}=0$ by Lindner (2022a) will not provide a good estimate of the susceptibility in this case, even if the refractory period is small compared to the mean ISI.

Intracellular recordings of a neuron’s voltage will not be limited to subthreshold values but will also include the action potentials. We showed that we can also include a (stereotypical) action potential in the voltage time series and demonstrated how this would change the FRR. We tested this for an alpha-function-shaped action potential added to the subthreshold voltage trace during the refractory period following each spike. We found again excellent agreement in the case of a white-noise driven IF model, in which our FRR is exact.

We also developed an approximate FRR for an IF neuron with correlated intrinsic noise. The approximation works well if the correlation time of the noise is significantly smaller than the mean ISI. In this case, we could use the FRR to estimate the intrinsic noise spectrum; again as a test, using here other versions of the FRR led to inaccurate estimates of this noise spectrum.

As an open problem remains to develop methods for the derivation of FRRs for the case of a slow background noise (as it may emerge in recurrent networks with synapses of intermediate strength (Ostojic 2014; Wieland et al. 2015)) or a narrow-band noise (Bauermeister et al. 2013), for which the correlation time might be equal or larger than the mean ISI. Another possible extension is to take into account spike-frequency adaptation (Benda and Herz 2003) and to derive an FRR for an IF model with an absolute and an additional variable for a spike-triggered adaptation (Brette and Gerstner 2005)—this kind of model has been very successful in reproducing and predicting spike trains of pyramidal cells for a given in vivo like noisy input current (Jolivet et al. 2008). Finally, because noise is not always Gaussian and additive but comes as a shot noise (Hohn and Burkitt 2001; Richardson and Swarbrick 2010; Droste and Lindner 2017) and, as a conductance modulation, has a substantial multiplicative component (Richardson and Gerstner 2005; Lindner and Longtin 2006; Wolff and Lindner 2008), it would be worth to explore how FRRs could be derived in this case. For the incorporation of shot noise a generalization of the Furutsu–Novikov theorem for such a non-Gaussian noise case is needed; the case of multiplicative noise in turn leads to higher-order correlation functions similar to those explored above.

Generally, it is interesting to note that the IF model has most recently also found application as a model of calcium spiking in non-neuronal cells (Ramlow et al. 2023a, b) and hence our results might also find applications to the problem of calcium signaling in cells. Calcium spikes encode signals (concentration variations of extracellular agonists binding to receptors in the cell membrane) with different timescales than typical neuronal signals (Friedhoff et al. 2021). However, relations between the spontaneous fluctuations and the response to stimuli are here of interest for the very same reasons as they are for neurons.

We would like to highlight the usefulness of the FRR by summarizing once more its possible applications. First of all, as in traditional statistical physics applications, we may use the spontaneous activity of the system to predict how it will respond to a time-dependent current stimulus—a characteristics that is of particular importance for signal encoders like neurons. Second, we may be able to derive novel analytical expressions for certain statistics such as the cross-spectrum between membrane voltage and spike train. Thirdly, in the case of colored noise, as we demonstrated in Fig. 5, we may be able to extract otherwise inaccessible statistics of the system such as the power spectrum of the intrinsic noise from accessible power and cross-spectra in absence and presence of a stimulus. Fourthly, given a certain neuron that we may assume to behave like an IF model, imposing the validity of the FRR gives us an independent criterion to fit model parameters (such that the FRR is, at least approximately, satisfied). Last but not least, the FRR imposes constraints on the neural information transfer because one of its characteristics, the signal-to-noise ratio for a weak stimulus signal has the susceptibility in the numerator and the spontaneous power spectrum of the spike train in the denominator—two quantities that are related via the FRR. The implications of this constraint are far from clear but potentially highly relevant to better understand limitations of neural signal transmission. In conclusion, there is plenty of motivation to further explore relations between spontaneous fluctuations and response characteristics of neurons.

## Figures and Tables

**Fig. 6:**
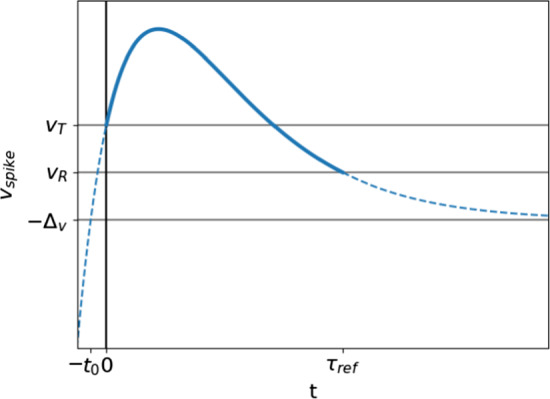
Spike shape with illustration of some parameters. We highlight the interval of the function $[0,\tau _\text {ref}]$, which is used in the IF model (dashed parts outside this range will be irrelevant to the model). For better visibility we choose parameters of the spike shape that are different to those in the first two figures in the main text: $\kappa =100, \Delta v=1, t_0=0.02, \beta =11.552$; here $t_0$ and $\beta$ were determined via the above formulas and the standard parameters $v_T=1,v_R=0$ and $\tau _{\text {ref}}=0.3$

## 6 Appendix

### 6.1 Parameters for the abstract spike shape

We recall that any spike shape $v_{\text {spike}}(t)$ has to obey the boundary conditions $v_{\text {spike}}(0)=v_T$ and $v_{\text {spike}}(\tau _{\text {ref}})=v_R$. For our abstract example $v_{\text {spike}}(t)=\kappa (t+t_0)e^{-\beta t}-\Delta v$, this conditions two of the parameters

40 $$\begin{aligned} t_0&=\frac{(v_T+\Delta v)}{\kappa } \end{aligned}$$

41 $$\begin{aligned} \beta&=\frac{1}{\tau _{\text {ref}}}\ln \left( \frac{\kappa \tau _{\text {ref}}+v_T+\Delta v}{v_R+\Delta v}\right) \end{aligned}$$

Furthermore, $\Delta v$ has to be selected such that $v_R+\Delta v>0$ and the dependent parameters are well defined. The other parameter $\kappa$ can then be chosen freely to define the peak height (larger with increasing $\kappa$) and peak timing (earlier with increasing $\kappa$). An example of the function with an illustration of some parameters is given in Fig. 6.

### 6.2 Evaluating the exact FRR for the LIF model with an abstract spike shape

The only remaining unknown in Eq. (24) is

42 $$\begin{aligned} \mathcal {F}\left( I_{\text {spike}}^{\text {LIFM}}\right) (\omega )=&\ \mathcal {F} (B_{\tau _{\text {ref}}}[\mu -v_\text {spike}-\dot{v}_{\text {spike}}])(\omega )\nonumber \\ =&\ \mu \tilde{B}_{\tau _{\text {ref}}}(\omega )+v_T-v_Re^{i\omega \tau _{\text {ref}}}\nonumber \\&-[1-i\omega ]\mathcal {F}(B_{\tau _{\text {ref}}}v_{\text {spike}})(\omega ) \end{aligned}$$

where again

43 $$\begin{aligned} \tilde{B}_{\tau _{\text {ref}}}(\omega )=\mathcal {F}(B_{\tau _{\text {ref}}})(\omega ) =\frac{1}{i\omega }\left[ e^{i\omega \tau _{\text {ref}}}-1\right] . \end{aligned}$$

For the abstract spike shape $v_{\text {spike}}(t)$, Eq. (8), we obtain

44 $$\begin{aligned}&\mathcal {F}(B_{\tau _{\text {ref}}}v_{\text {spike}})(\omega ) =\ \kappa \Bigg [\left[ \frac{1}{(\beta +i\omega )^2}-\frac{t_0}{\beta +i\omega }\right] \nonumber \\&-\left[ \frac{1}{(\beta +i\omega )^2}+\frac{\tau _{\text {ref}}-t_0}{\beta +i\omega }\right] e^{-(\beta +i\omega )\tau _{\text {ref}}}\Bigg ]-\Delta v\tilde{B}_{\tau _{\text {ref}}}(\omega ). \end{aligned}$$

### 6.3 Rewriting averages involving the spike train *x*

In this section, we derive two identities for averages involving the spike train *x*. We employ the following regularization of the involved delta distributions

45 $$\begin{aligned} x(t):=\lim _{\Delta t\rightarrow 0}\frac{n(t)}{\Delta t} \end{aligned}$$

where *n*(*t*) is the spike indicator function, which depicts whether there is at least one spike within a time bin of size $\Delta t$ centered around *t*

46 $$\begin{aligned} n(t)= {\left\{ \begin{array}{ll} 1,&{}\text { if }\exists \ i\ \epsilon \ I_s \text { with }t_i\ \epsilon \ [t-\frac{\Delta t}{2}, t+\frac{\Delta t}{2}]\\ 0,&{}\text { otherwise}. \end{array}\right. } \end{aligned}$$

Here $I_s$ is the index set of spike times.

We start with the identity for the triple correlation, Eq. (20), first mentioned in Sec. 2 that we restate here

47 $$\begin{aligned} \langle x(t_1)x(t_2)\eta (t_3)\rangle =[C_{xx}(t_1-t_2)+r_0^2]\langle \eta (t_3)\rangle _{t_1,t_2} \end{aligned}$$

We rewrite this average as follows:

48 $$\begin{aligned}&\hspace{-1em}\langle x(t_1)x(t_2)\eta (t_3)\rangle \nonumber \\ =&\lim _{N\rightarrow \infty }\frac{1}{N}\sum _{\ell \ \in \ I}x_\ell (t_1)x_\ell (t_2)\eta _\ell (t_3)\nonumber \\ =&\lim _{N\rightarrow \infty }\lim _{\Delta t\rightarrow 0}\frac{1}{N}\sum _{\ell \ \in \ I}\frac{n_\ell (t_1)}{\Delta t}\frac{n_\ell (t_2)}{\Delta t}\eta _\ell (t_3)\nonumber \\ =&\lim _{N\rightarrow \infty }\lim _{\Delta t\rightarrow 0}\frac{1}{N}\frac{1}{\Delta t^2}\sum _{\ell \ \in \ I_{t_1,t_2}}\eta _\ell (t_3)\nonumber \\ =&\lim _{N\rightarrow \infty }\lim _{\Delta t\rightarrow 0}\frac{1}{N}\frac{1}{\Delta t^2}N_{t_1,t_2}\frac{1}{N_{t_1,t_2}}\sum _{\ell \ \in \ I_{t_1,t_2}}\eta _\ell (t_3)\nonumber \\ =&\lim _{N\rightarrow \infty }\lim _{\Delta t\rightarrow 0}\frac{1}{N}\sum _{\ell \ \in \ I}\frac{n_\ell (t_1)}{\Delta t}\frac{n_\ell (t_2)}{\Delta t}\frac{1}{N_{t_1,t_2}}\sum _{\ell \ \in \ I_{t_1,t_2}}\eta _\ell (t_3)\nonumber \\ =&\langle x(t_1)x(t_2)\rangle \langle \eta (t_3)\rangle _{t_1,t_2}\nonumber \\ =&[C_{xx}(t_1-t_2)+r_0^2]\langle \eta (t_3)\rangle _{t_1,t_2}. \end{aligned}$$

where we used $I_{t_1,t_2}$ and $N_{t_1,t_2}$ to denote the index set and number of realizations with spike times in time bins around $t_1$ and $t_2$ whereas *I* and *N* indicate the index set and number of *all* realizations.

Now, we derive the identity (used in the derivation of Eq. (33))

49 $$\begin{aligned} \langle \eta (t_2)\rangle _{t_1}=\frac{1}{r_0}\langle x(t_1)\eta (t_2)\rangle \end{aligned}$$

Let $I_{t_1}$ and $N_{t_1}$ denote the index set and number of realizations with spikes within a time bin of size $\Delta t$ centered in $t_1$ (both possess an implicit dependence on $\Delta t$). Then

50 $$\begin{aligned} \hspace{0em}\langle \eta (t_2)\rangle _{t_1}\nonumber \\ =&\lim _{N_{t_1}\rightarrow \infty }\lim _{\Delta t\rightarrow 0}\frac{1}{N_{t_1}}\sum _{r\ \epsilon \ I_{t_1}}\eta _r(t_2)\nonumber \\ =&\lim _{N_{t_1}\rightarrow \infty }\lim _{\Delta t\rightarrow 0}\frac{1}{N_{t_1}}\sum _{r\ \epsilon \ I_{t_1}}n_r(t_1)\eta _r(t_2)\nonumber \\ =&\lim _{N\rightarrow \infty }\lim _{\Delta t\rightarrow 0}\frac{N\Delta t}{N_{t_1}}\frac{1}{N}\sum _{r\ \epsilon \ I}\frac{n_r(t_1)}{\Delta t}\eta _r(t_2)\nonumber \\ =&\lim _{N\rightarrow \infty }\lim _{\Delta t\rightarrow 0}\frac{N\Delta t}{\sum _{r'\ \epsilon \ I}n_{r'}(t_1)}\frac{1}{N}\sum _{r\ \epsilon \ I}\frac{n_r(t_1)}{\Delta t}\eta _r(t_2)\nonumber \\ =&\lim _{N\rightarrow \infty }\lim _{\Delta t\rightarrow 0}\frac{1}{\frac{1}{N}\sum _{r'\ \epsilon \ I}\frac{n_{r'}(t_1)}{\Delta t}}\frac{1}{N}\sum _{r\ \epsilon \ I}\frac{n_r(t_1)}{\Delta t}\eta _r(t_2)\nonumber \\ =&\frac{1}{\langle x(t_1)\rangle }\langle x(t_1)\eta (t_2)\rangle =\frac{1}{r_0}\langle x(t_1)\eta (t_2)\rangle \end{aligned}$$

where we used again in the last line that the unperturbed spike train is stationary.

### 6.4 The conditional noise average for small refractory periods

We show that

51 $$\begin{aligned} \langle \eta \rangle _{-\tau '}\approx \langle \eta \rangle _{0},\quad 0<\tau '<\tau _{\text {ref}} \end{aligned}$$

as long as

52 $$\begin{aligned} C_{\eta \eta }(\tau +\tau ')\approx C_{\eta \eta }(\tau ),\quad \tau >0 \end{aligned}$$

for any $\tau '\ \epsilon \ [0,\tau _{\text {ref}}]$. This then implies

53 $$\begin{aligned} \langle \eta \rangle _{\text {ref}}:=\frac{1}{\tau _{\text {ref}}} \int \limits _0^{\tau _{\text {ref}}}d\tau ''\langle \eta \rangle _{-\tau ''}\approx \langle \eta \rangle _{\tau '},\quad 0<\tau '<\tau _{\text {ref}} \end{aligned}$$

as claimed in Sec. 4.

54 $$\begin{aligned} \langle \eta \rangle _{-\tau '}&=\frac{1}{r_0}\langle x(-\tau ')\eta (0)\rangle \nonumber \\&=\frac{1}{r_0}\int \limits _{-\infty }^{\infty }d\tau \ C_{\eta \eta }(\tau +\tau ')K_x(\tau )\nonumber \\&=\frac{1}{r_0}\int \limits _{0}^{\infty }d\tau \ C_{\eta \eta }(\tau +\tau ')K_x(\tau )\nonumber \\&\approx \frac{1}{r_0}\int \limits _{0}^{\infty }d\tau \ C_{\eta \eta }(\tau )K_x(\tau )=\langle \eta \rangle _{0} \end{aligned}$$

where we used the Furutsu–Novikov theorem (line two to line three) and the assumption on the autocorrelation function of the noise process, Eq. (52) (line three to line four).
